# Pancoast's Syndrome due to Fungal Abscess in the Apex of Lung in an Immunocompetent Individual: A Case Report and Review of the Literature

**DOI:** 10.1155/2014/581876

**Published:** 2014-09-11

**Authors:** Anirban Das, Sabyasachi Choudhury, Sumitra Basuthakur, Sibes Kumar Das, Angshuman Mukhopadhyay

**Affiliations:** Department of Pulmonary Medicine, Medical College, Kolkata, West Bengal, India

## Abstract

Malignant tumours in the apices of the lungs, especially bronchogenic carcinoma (Pancoast tumours), are the most common cause of Pancoast' syndrome which presents with shoulder or arm pain radiating along the medial aspect of forearm and weakness of small muscles of hand with wasting of hypothenar eminence due to neoplastic involvement of C8 and T1 and T2 nerve roots of brachial plexus. There are a number of benign conditions which may lead to Pancoast's syndrome; fungal abscess located in the apex of lung is one of them. Oral or intravenous antifungals are the treatment of choice in this case and complete recovery is usual, whereas, surgical resection followed by chemoradiotherapy is the treatment of choice in case of Pancoast's syndrome due to lung cancers. Hence, tissue diagnosis is mandatory. Here, we report a case of apical fungal abscess causing Pancoast's syndrome in an immunocompetent individual of 35 years of age to raise the awareness among the clinicians regarding this rare clinical entity.

## 1. Introduction

Pancoast's syndrome is characterized by the pain in superior extremity and weakness and wasting of the small muscles of hand. Sometimes, it may be associated with ipsilateral Homer's syndrome. In 1924, it was first described by Henry Pancoast [1, 2]. This syndrome is most commonly caused by a malignant tumour located at the superior pulmonary sulcus of the thorax, known as Pancoast tumour. Squamous cell carcinoma of lung is the most common histological type causing Pancoast's syndrome [3]. Bronchogenic carcinoma is responsible for over 80% of cases [4]. Other malignant causes of Pancoast's syndrome are non-Hodgkin's lymphoma, pleural mesothelioma, multiple myeloma, solid tumour metastases (liver, cervix, urinary bladder, and kidneys), adenoid cystic carcinoma, malignant neurogenic tumours, thyroid carcinoma, and so forth [3, 5, 6]. Benign causes of Pancoast's syndrome are rarely reported in the literature. Here, we report a case of fungal abscess located in the apex of lung causing Pancoast's syndrome in an immunocompetent housewife of thirty-five years of age.

## 2. Case Report

A thirty-five-year-old nonsmoker, nondiabetic female presented with low grade, intermittent fever and a shooting type of pain in left shoulder and arm radiating through medial aspect of left forearm and hand for 4 months. The pain was gradually progressive and was associated with weakness and wasting of hypothenar muscles. Severity of the shoulder pain was more at night, and it was not relieved by simple analgesic. There was history of cough and scanty, mucoid expectoration for the same duration. There was no history of shortness of breath, hemoptysis, and heaviness of the chest. History of anorexia, significant weight loss, night sweats, and fatigue were present.

**Figure 1:**
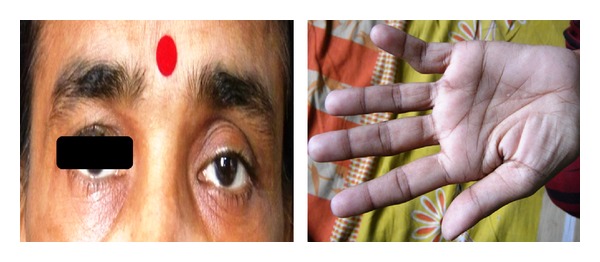
Photograph showing left sided partial ptosis and enophthalmos with wasting of hypothenar eminence of left hand.

General examination revealed anemia, but no clubbing, and enlarged superficial lymph node. Her pulse rate was 100 beats/minute, respiratory rate 16 breaths/minute, temperature 100°F, and blood pressure 110/70 mmHg. Examination of eye revealed left sided Horner's syndrome only, that is, presence of partial ptosis, enophthalmos, miosis, in association with anhidrosis over the left hemifacial region, and loss of ciliospinal reflex with preservation of pupillary light reflex and corneal reflex on the same side (Figure 1). Examination of left superior extremity revealed only the weakness of small muscles of the left hand (grade III power) and wasting of hypothenar eminence on left side (Figure 1). Examination of respiratory system revealed central mediastinum and dull percussion note over clavicles, second and third intercostal spaces over midclavicular line, and suprascapular areas on both sides. There were diminished vesicular breath sounds and decreased vocal resonance over both infraclavicular and suprascapular areas. Examination of other systems did not reveal any abnormality.

**Figure 2:**
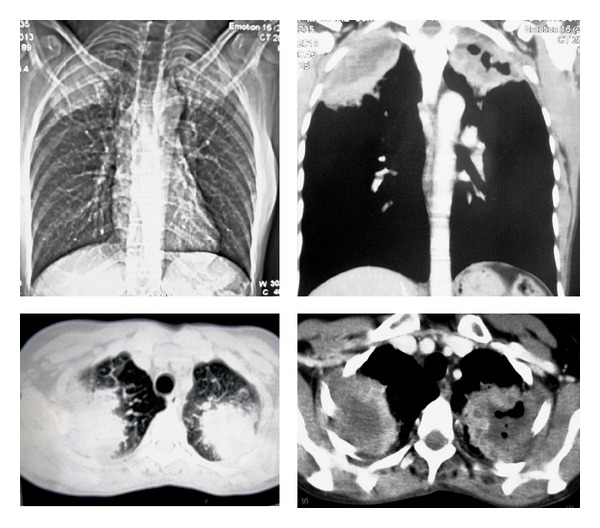
CECT thorax showing heterogeneous, pleural based lung masses in upper lobes of both sides.

**Figure 3:**
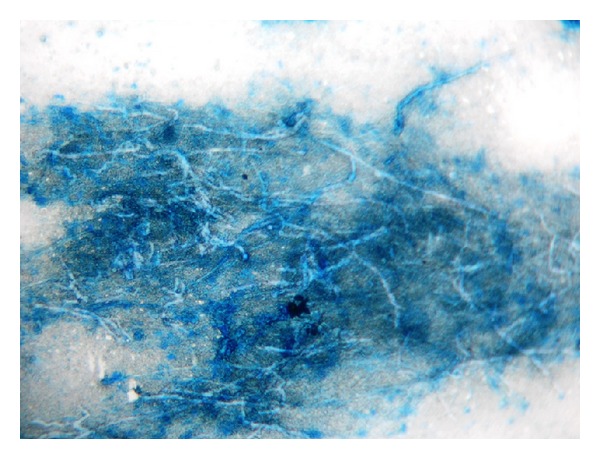
Microphotograph of histopathological examination of CT-guided tru-cut biopsy showing branching filamentous fungi with septate hyphae with finger-like branching at acute angles (lactophenol cotton blue stain, 40x).

**Figure 4:**
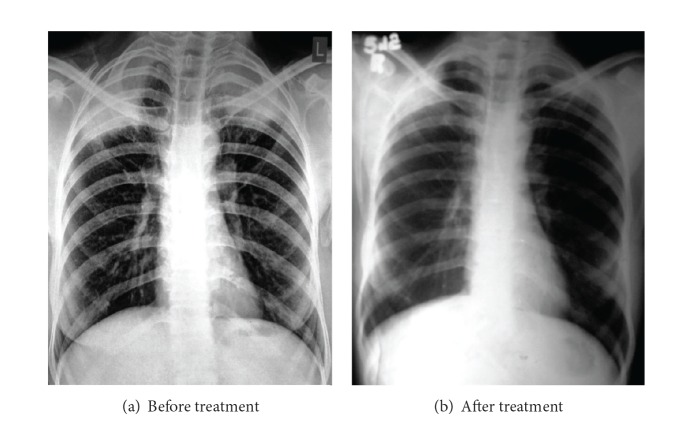
CXR-P.A. views showing pretreatment bilateral apical consolidations with necrosis (a) and posttreatment almost complete resolution of the lesions (b).

Complete hemogram and blood biochemistry were within normal limit, except that hemoglobin concentration was 8.3 g/dL. Blood for anti-HIV-1 and anti-HIV-2 antibodies was negative. Spontaneous and induced sputum for acid fast bacilli and malignant cells was negative. Mantoux test (5 TU) was positive (11 mm induration), indicating integrated cell mediated immunity. Chest X-ray-posteroanterior view (P.A. view) showed bilateral pleural based alveolar air space consolidation in upper zone. Sputum for fungal smear was negative. Contrast enhanced computed tomography (CECT) scan of thorax showed two ill-defined, heterogeneous, mildly enhanced, partly necrotic, pleural based lesions in the apicoposterior segments of both upper lobes (Figure 2). There was no rib erosion. CT-guided fine needle aspiration cytology (FNAC) from both lesions showed suppurative inflammation and no acid fast bacilli, and malignant cell was detected. Gram stain, pyogenic culture, fungal smear, and mycobacterial culture of FNAC materials were negative. Fibreoptic bronchoscopy revealed no endobronchial lesion and bronchoalveolar lavage (BAL) fluid did not show any abnormality. Finally, CT-guided tru-cut biopsy tissue taken from the lesions of both sides showed branching filamentous fungi with septate hyphae branching at acute angles (lactophenol cotton blue stain), suggestive of bilateral fungal abscesses in upper lobes, out of which the left one resulted in Pancoast's syndrome (Figure 3). Finally, fungal culture of biopsy tissue showed growth of fungus with septate hyphae having finger-like branching at acute angles, suggestive of growth of* Aspergillus fumigatus*. Hence, the diagnosis was bilateral upper lobe lung abscesses due to* Aspergillus*, out of which the left one causes Pancoast's syndrome. The patient was treated with itraconazole tablet, 200 mg (two tablets, each of 100 mg) twice daily for six weeks. Six weeks of treatment with oral antifungals resulted in radiological resolution of both apical lesions (Figure 4), along with clinical recovery of left sided Pancoast's syndrome and Horner's syndrome, documented on followup.

## 3. Discussion

Pancoast's syndrome is characterized by shoulder and arm pain which is radiated to ulnar aspect of arm and forearm, Claude Bernard-Horner's syndrome, and weakness and atrophy of ipsilateral hypothenar muscles. Destruction of adjacent vertebral bodies or first, second, and third ribs with dense white apical shadow is detected on chest radiograph. It is due to involvement of C8 and T1 and T2 nerve roots of brachial plexus (lower brachial plexopathy) and paravertebral cervical sympathetic trunk above the stellate ganglion. Excruciating chest pain may be due to erosion of ribs and anterior chest wall. Pancoast tumours, that is, apical lung cancers and other malignancies, are predominant causes of Pancoast's syndrome. Besides them, few benign tumours like solitary pleural fibroma and infective conditions like fungal abscess caused by* Aspergillus, Cryptococcus, Mucor*, or* Allescheria boydii*, apical tuberculosis, hydatid cyst, and bacteria (e.g.,* Staphylococci, Pseudomonas, Actinomyces*, and* Nocardia*) are reported to cause Pancoast's syndrome in the literature [7–9].

Immunosuppressed patients (diabetes, HIV infection, congenital immunodeficiency, postchemotherapy, neutropenia, etc.) are susceptible to invasive fungal infections which may cause Pancoast's syndrome due to direct or vascular invasion of bones, soft tissues, and nerves at thoracic inlet [10]. But, surprisingly, in our case bilateral apical* Aspergillus* abscesses were seen of which the left one was producing Pancoast's syndrome without any evidence of immunosuppression.

CECT thorax or magnetic resonance imaging of neck is essential to demonstrate anatomical details of the lesion and the locoregional extension of the lesion into the surrounding soft tissues, especially that of brachial plexus [11].

Fungal staining of sputum smear and fungal culture of sputum or bronchial washing obtained by fibreoptic bronchoscopy is frequently negative due to peripheral location of the infiltrate. Integrated defense mechanisms of lung parenchyma contain the infection in immunocompetent individuals, thus making the isolation of fungus from sputum and bronchial aspirate difficult. So in this situation, CT-guided tru-cut biopsy of peripherally located fungal abscess is essential to detect* Aspergillus* infection.* Aspergillus* hyphae are narrow with septate branches at 45° angles. Finally, fungal culture of the biopsy material shows the growth of* Aspergillus* to confirm the diagnosis as in our case.

Pancoast's syndrome due to fungal etiology can successfully be treated by antifungals like oral itraconazole or voriconazole [12]. Intravenous amphotericin B is an alternative [12]. Surgical excision followed by chemoradiotherapy is the standard treatment for Pancoast's syndrome, caused by superior sulcus tumours, and prognosis is definitely guarded. Hence, tissue diagnosis by image-guided tru-cut biopsy is essential in any case of Pancoast' syndrome to confirm the etiology, as reversible etiology like fungal abscess is associated with complete resolution of the syndrome by administration of antifungals only.

## References

[1] Pancoast HK (1924). Importance of careful roentgen-ray investigationsof apical chest tumors. *The Journal of the American Medical Association*.

[2] Pancoast H (1932). Superior pulmonary sulcus tumor: tumor characterizedby pain, Horner’s syndrome, destruction of bone andatrophy of hand muscles. *The Journal of the American Medical Association*.

[3] Archie VC, Thomas CR (2004). Superior sulcus tumors: a mini-review. *The Oncologist*.

[4] Comet R, Monteagudo M, Herranz S, Gallardo X, Font B (2006). Pancoast's syndrome secondary to lung infection with cutaneous fistulisation caused by Staphylococcus aureus. *Journal of Clinical Pathology*.

[5] Chang C-F, Su W-J, Chou T-Y, Perng R-P (2001). Hepatocellular carcinoma with Pancoast's syndrome as an initial symptom: a case report. *Japanese Journal of Clinical Oncology*.

[6] Panagopoulos N, Leivaditis V, Koletsis E (2014). Pancoast tumors: characteristics and preoperative assessment. *Journal of Thoracic Disease*.

[7] Fibla JJ, Penagos JC, León C (2004). Pseudo-pancoast syndrome caused by a solitary fibrous tumor of the pleura. *Archivos de Bronconeumologia*.

[8] White HD, White BAA, Boethel C, Arroliga AC (2011). Pancoast’s syndrome secondary to infectious etiologies: A not so uncommon occurrence. *The American Journal of the Medical Sciences*.

[9] Comet R, Monteagudo M, Herranz S, Gallardo X, Font B (2006). Pancoast's syndrome secondary to lung infection with cutaneous fistulisation caused by *Staphylococcus aureus*. *Journal of Clinical Pathology*.

[10] Bansal M, Martin SR, Rudnicki SA, Hiatt KM, Mireles-Cabodevila E (2011). A rapidly progressing Pancoast syndrome due to pulmonary mucormycosis: a case report. *Journal of Medical Case Reports*.

[11] Manenti G, Raguso M, D'Onofrio S (2013). Pancoast tumor: the role of magnetic resonance imaging. *Case Reports in Radiology*.

[12] Vahid B, Marik PE (2007). Fatal massive hemoptysis in a patient on low-dose oral prednisone: chronic necrotizing pulmonary aspergillosis. *Respiratory Care*.

